# Valproic Acid Enhances Reprogramming Efficiency and Neuronal Differentiation on Small Molecules Staged-Induction Neural Stem Cells: Suggested Role of mTOR Signaling

**DOI:** 10.3389/fnins.2019.00867

**Published:** 2019-09-04

**Authors:** Qingrui Duan, Siyi Li, Xinrui Wen, Gavin Sunnassee, Jian Chen, Sheng Tan, Yang Guo

**Affiliations:** Department of Neurology, Zhujiang Hospital, Southern Medical University, Guangzhou, China

**Keywords:** neural stem cells, VPA, mTOR signaling, small molecule, reprogramming, stage-induction

## Abstract

Inducing somatic cells into neural stem cells (iNSCs) in specific ways provides a new cell therapy in a variety of neurological diseases. In the past, iNSCs were generated by transcription factors which increased the risk of mutagenesis, tumor formations, and immune reactions by viral transduction vectors. Therefore, in this study, different small molecules were used to induce mouse embryonic fibroblasts (MEFs) into iNSCs in different reprogramming stages, which showed high reprogramming efficiency without altering the genome. We demonstrated that the small molecules staged-induction neural stem cells (SMSINS) have the characteristics of neural stem cells (NSCs) in morphology, gene expression, self-renewal and differentiation potential. Furthermore, valproic acid (VPA), one of small molecules, was showed to enhance neural induction with highest efficiency compared with six other small molecules, which were also investigated in the present study. Moreover, our results suggested that activating the mammalian target of rapamycin (mTOR) signaling enhanced the induction efficiency and neuronal differentiation. Collectively, our findings indicated that using this induction program allowed us to obtain safe and efficient iNSCs which were free of genetic manipulation. The VPA-mediated mTOR signaling pathway may enhance reprogramming efficiency and neuronal differentiation. So we suggested that this program could be a new method of obtaining iNSCs for the treatment of neurological diseases by cell replacement therapy in the future.

## Introduction

Cellular replacement holds potential opportunities to treat diseases which result from loss of specific cells like NSCs in neurodegenerative diseases such as ischemic stroke, Parkinson’s disease, and spinal cord injury (Hedlund and Perlmann, 2009). NSCs could be generated from induced differentiation of iPSCs or ESCs (Smith et al., 2008; Chambers et al., 2009). Takahashi et al. (2007) illustrated that iPSCs could be directly generated from mouse or human fibroblasts by the additional supplements of defined transcription factors (Takahashi and Yamanaka, 2006). However, iPSCs need to be differentiated into specific-lineage cell types for cell therapeutic purpose with long induction period and low induction efficiency. Moreover, iPSCs may cause teratomas, a major safety hurdle in clinical settings (Fong et al., 2010). Thus, somatic cells could be directly reprogrammed into lineage-restricted stem cells such as NSCs, which enhances differentiation efficiency and lowers the risk of teratoma formation (Ring et al., 2012). Compared with iPSCs, iNSCs not only maintain its proliferative potential, but also differentiate more easily into three neuronal lineages, namely neurons, astrocytes, and oligodendrocytes. Thus, iNSCs are more suitable than iPSCs in clinical applications for the treatments of neurological diseases.

Recently, some researchers found fibroblasts could be reprogrammed into NSCs using transcription factors (Han et al., 2012; Ring et al., 2012; Thier et al., 2012). However, they were limited to clinical applications because of the risk of viral transduction vectors and the transgene integration. To find a way without the insertion of any gene, several studies have focused on small molecules to regulate signaling pathways and improve reprogramming efficiency (Cheng et al., 2014; Han et al., 2016; Zhang et al., 2016; Zheng et al., 2016). VPA, as one small molecule, shows a direct inhibition of HDACs inhibitors activity and causes hyperacetylation of histones (Phiel et al., 2001). In the CNS, VPA promoted the neurogenesis of embryonic rat cortical cells to striatal primordial stem cells, and increased neuronal differentiation of NSCs (Laeng et al., 2004). Studies have shown that VPA not only can improve reprogramming efficiency (Cheng et al., 2014; Mirakhori et al., 2015; Han et al., 2016), but also induce neuronal differentiation of adult hippocampal NSCs and inhibit astrocytes and oligodendrocytes differentiation (Yu et al., 2009; Zhang et al., 2017). In addition, VPA exposure also activated mTOR signaling which is a key regulator to proliferation in NSCs or NPCs and essential for terminal differentiation of NSCs (Hsieh et al., 2004).

In the preparation phase, we found that the reprogramming efficiency and neuronal differentiation exhibited significant differences with or without VPA. We hypothesized that exposure to VPA could enhance induced efficiency and neuronal differentiation through activating mTOR signaling. Then we discussed the effect of specific mTOR signaling activators or inhibitors on reprogramming efficiency to find a new pathway in the induction program.

## Materials and Methods

### Ethics Statement

This study was carried out in accordance with the National Institutes of Health guide for the care and use of Laboratory animals (NIH Publications No. 8023, revised 1978). The protocol was approved by the Bioethics Committee of Southern Medical University, Guangzhou, China.

### Mouse Embryonic Fibroblasts Preparation and Mouse Neural Stem Cells

Isolation of MEFs was from E13.5 mouse embryos (C57BL/6, stain) as (Zheng et al., 2016). To remove neural tissues and derive pure fibroblasts, the head, limb, tail, and internal organs were removed from the E13.5 mouse embryos. Then the body tissues were slices into small pieces, trypsinized, and culture in MEFs medium [DMEM (Gibco) supplement with 10% FBS (Gibco) 1% penicillin/streptomycin (Gibco)] at 37°C with 5% CO_2_. In this study, we used MEFs with the third passage for avoiding cellular senescence. After separating the bodies of embryos from the heads, the cerebral cortexes were separated from heads with stereomicroscope, then cut up and were digested with 0.05% trypsin to obtain single cells of NSCs that cultured in NSCs medium [DMEM/F12 (Gibco) containing with 1 × N2 supplement (Gibco), 1 × B27 supplement (Gibco), 2 mM L-Glutamine (Gibco), 20 ng/ml EGF (Peprotech), and 20 ng/ml bFGF (Peprotech)].

### Stage-Induction of SMSINS Cells

Generally, MEFs were treated with LDN193189 (selleck S2618, 0.25 μM), SB431542 (selleck S1067, 1 μM) on day 1 and day 2. CHIR99021 (selleck S2924, 3 μM), DAPT (selleck S2215, 5 μM), and VPA (sigma P4543, 0.5 mM) were used on day 3 and day 4. CHIR99021 (selleck S2924,3 μM), DAPT (selleck S2215, 5 μM) were used on day 5 and day 6. Shh (Peprotech 315225, 100 ng/ml) and Purmorphamine (selleck S3042, 1 μM) were used to improved neural differentiation on day 7 and day 8. Then, all small molecules were withdrawn on the last 2 days. After 10 days, SMSINS cells were plate into coated poly-D-lysine (10 μg/ml)/laminin (5 μg/ml) (sigma) (PDL/L) for proliferation or next test. However, for neurosphere culture, SMSINS cells were trypsinized to single cells and seeded into uncoated 24 well-plates in NSC medium. The half of medium was switched every 2 days.

To mediate mTOR signaling, we repeated the program except that Rapamycin (sigma V900930, 2 μM) was added on day 3 and day 4 and MHY1485 (selleck S7811, 1 μM) replaced VPA.

### Differentiation of SMSINS Cells *in vitro*

Cells were seeded at 5000 onto PDL/L 24 well-plates in special medium (Zheng et al., 2016). For spontaneous differentiation, cells were changed to NSCs medium without containing bFGF and EGF, named N2-B27 medium, for 2 or 3 weeks. For neuron differentiation, cells were cultured in Neural basal medium [50% Neurobasal Plus Medium (Gibco) and 50% DMEM/F12 containing with 1 × N2 supplement, 1 × B27 supplement, 2 mM L-Glutamine] supplement with 10 ng/ml BDNF (Peprotech) and 200 μM ascorbic acid (Sigma) for at least 2 weeks. As for mature neuron differentiation, the medium was switched neuron medium containing NSCs medium supplement with 100 ng/ml Shh and 1 uM retinoic acid (sigma) for 4 days, then replaced with the Neural basal medium supplement with 10 ng/ml BDNF, 10 ng/ml GDNF (Peprotech), 2 μM cAMP (sigma), and 200 μM ascorbic acid for 4 weeks. To differentiate into astrocytes, the special astrocyte medium used was a mixture of NSC medium and 20 ng/ml CNTF (Peprotech) for 4 days. Then all growth factors were withdrawn and replaced with 10 ng/ml BDNF, 10 ng/ml GDNF, and 200 μM ascorbic acid for the next 14 days. For oligodendrocytes differentiation, SMSINS were cultured in NSCs medium supplement with 10 ng/ml NT-3 (Peprotech) and 20 ng/ml PDGF (Peprotech) for 6 days, then N2-B27 medium containing with 10 ng/ml NT-3, 20 ng/ml PDGF, and 200 μM ascorbic acid for 8 days. For further differentiation of the SMSINS cells into mature oligodendrocytes, 10 ng/ml BDNF and 10 ng/ml GDNF were added to N2-B27 medium containing 200 μM ascorbic acid for at least 2 weeks.

To differentiate into neuronal subtypes, dopaminergic neuron, cells were cultured into NSCs medium supplement with 100 ng/ml FGF8 (Peprotech) and 200 ng/ml Shh for 4 days. Then the medium was switched the Neural basal medium containing with 10 ng/ml BDNF, 10 ng/ml GDNF, and 200 μM ascorbic acid for 14 days. For cholinergic neuron differentiation, the same conditions as dopaminergic neuron were used, except that in the latter case 1 μM retinoic acid and 100 ng/ml Shh were added to NSC medium for the first 4 days instead of FGF8 and 200 ng/ml Shh. GABAergic neuron medium was approximately identical to dopaminergic neuron, but FGF8 was replaced by 10 μM VPA for the first 4 days.

### Immunofluorescence

Cells were washed once with 1 × PBS and fixed in 4% paraformaldehyde (Leagene) for 15 min at room temperature. After washing three times with 1 × PBS, cells were permeabilized with 0.5% Triton X-100 in 1 × PBS for 15 min, washed three times with 1 × PBS, and blocked with 10% goat serum for at least 30 min. Then, all primary antibodies were diluted in 1 × PBS and incubated cells overnight at 4°C. Primary antibodies were anti-Nestin (Millipore, 1:100, rat), anti-Sox2 (abcam, 1:100, mouse), anti-Tuj1 (Santa cruz, 1:100, mouse), anti-NeuN (abcam, 1:300, rabbit), anti-GFAP (Santa cruz, 1:100, rabbit), anti-olig2 (Proteintech, 1:1000, rabbit), anti-TH (Santa cruz, 1:100, rabbit), anti-ChAT (abcam, 1:100, rabbit), anti-GABA (sigma, 1:100, mouse), anti-Nav 1.7 (abcam, 1:100, mouse), anti-COL1A1 (Bioss, 1:400, mouse), anti-Collagen IV (abcam, 1:250, rabbit), and anti-Oct4 (Bioss, 1:400, rabbit). Next, cells were washed three times with 1 × PBS and incubated with secondary antibodies FITC (bioss, 1:100) or Cy3 (cwbio, 1:100) for 30 min at room temperature, followed three 5 min-washed with 1 × PBS. The nuclei were stained with DAPI (BestBio, 1:100) for 15 min.

### CCK-8 Cell Assay

The CCK-8 assay (ApexBio, Cell Counting Kit-8) was used to quantitatively evaluate cell proliferation. Briefly, SMSINS were seeded in PDL/L 96-well plates with three replicates in each group. After cells attachment which was recorded as 0 h, 10 μL CCK-8 solutions were added to each well, and cells were incubated for 4 h. The absorbance at 450 nm was measured with a microplate reader (Thermo Fisher Scientific). Then cells of each group were examined at 12, 24, 48, 72, 96, and 120 h. Each experiment was repeated for three times.

### RNA Preparation and qRT-PCR

Total RNA was extracted from cells using Trizol (Invitrogen) and was converted to cDNA using All-in-one^TM^ First-cDNA Synthesis (GeneCopoeia) according to the guidelines from its manual. Quantitative PCR was performed 45 cycles using the primers listed at Supplementary Table S1. Primer Information (Sangong Biotech) following the protocol of All-in-one^TM^ qPCR Mix (GeneCopoeia). All PCR was performed in triplicate and the housekeeping gene Glyceraldehyde 3-phosphate dehydrogenase (GAPDH) was used as an internal standard.

### Western Blot

The detailed steps of Western blotting have been described previously (Xuan et al., 2015). Simply put, cellular proteins were extracted with RIPA lysis buffer (cwbio) supplement with protease inhibitor cocktail (abcam) and phosphatase inhibitor cocktail (sigma P0044). Then, protein concentration was assessed with the BCA kit (Sangong Biotech). Protein samples were separated by SDS-PAGE and transferred to polyvinylidene difluoride membranes. Blotting membranes were incubated with 5% bovine serum albumin or 5% no-fat powdered milk in tris buffered saline with 1% tween and incubated primary antibodies mTOR (CST, 1:1000), P-mTOR (CST, 1:1000), and β-actin (abway, 1:10000). After incubation with horseradish peroxidasecoupled secondary antibodies for 2 h at room temperature, quantified by densitometry.

### Fluorescence-Activated Cell Sorting Analysis

Neural stem cells and SMSINS cells were seeded onto PDL/L dishes in NSC medium. Cells were dissociated and 1 × 10^5^ cells were transferred to Fluorescence-Activated Cell Sorting (FACS) tubes (BD Biosciences). Cells were rinsed twice with Flow Cytometry Staining Buffer, then added freshly prepared Fixation/Permeabilization Buffer for 40 min at 4°C in the dark and incubated at room temperature for 15 min in normal rat serum. Thereafter, cells were incubated with primary anti-Nestin (abcam, 1:50) and anti-Sox2 (CST, 1:50) antibodies for 40 min at 4°C in the dark, then added secondary antibodies at room temperature for 20 min. Cells were washed twice with Permeabilization Buffer and then resuspended in Flow Cytometry Staining Buffer before running FACS analysis.

### Statistical Analysis

All analyses were detected using SPSS 22.0 software (SPSS, Chicago, IL, United States). If data conform normal distribution and variance homogeneity, One-way ANOVA followed by Bonferroni’s *post hoc* test or Dunnett’s T3 was performed and Student’s *t*-test was used for comparisons of two groups. If data could not conform normal distribution or variance homogeneity, Non-parametric test was used to compare different groups. *p* values < 0.05 were considered significant.

## Results

### SMSINS Cells Can Be Obtained From MEFs by a Staged-Induction Program of Small Molecules

**FIGURE 1:**
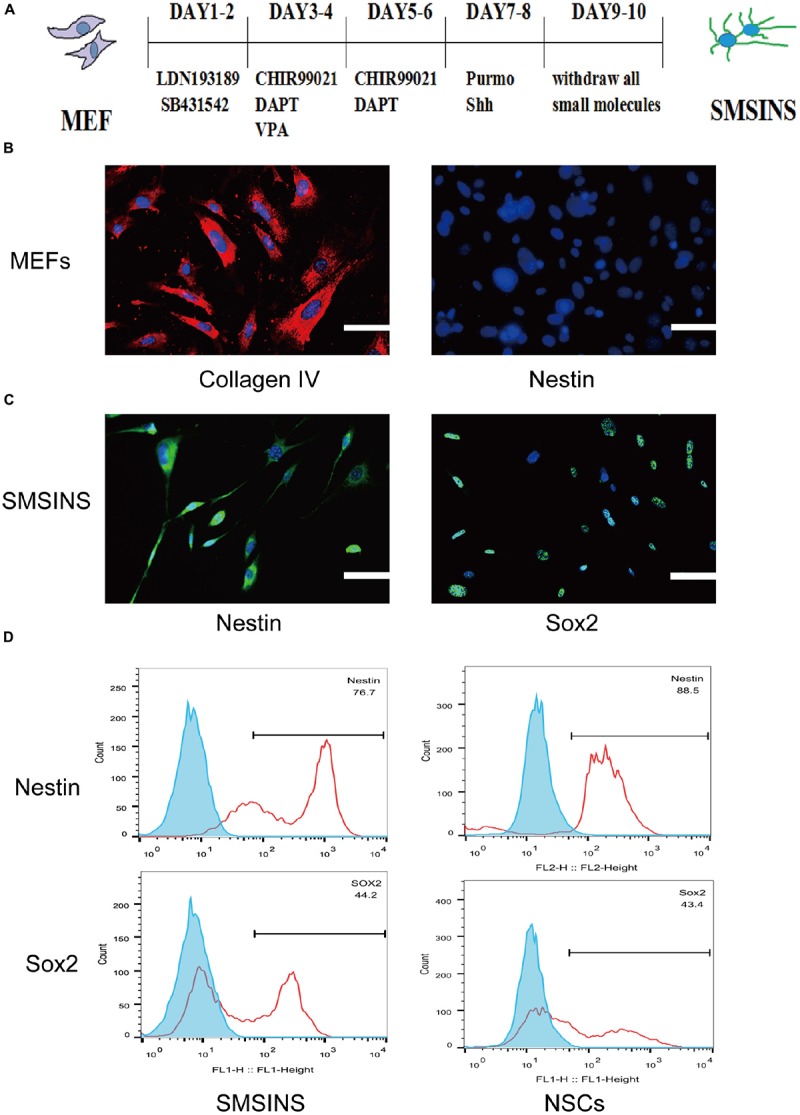
Induction of SMSINS from MEFs. **(A)** Schematic diagram of the SMSINS induced process. **(B)** MEFs express fibroblast markers without NSC markers. MEFs were stained with specific antibodies against Collagen IV (Red) and Nestin (Green). **(C)** Immunostaining of Nestin (Green) and Sox2 (Green) in SMSINS at day 10 using a specific antibody. Nuclei were counterstained with DAPI (blue). **(D)** Flow cytometric analysis to quantify cells expressing Sox2 and Nestin after SMSINS induction and native NSC cells isolated from embryonic mouse brain. Scale bars: 100 μm **(B,C)**.

To investigate whether small molecules can replace transcription factors to chemically reprogram MEFs into NSCs, we searched the literature broadly to identify potential candidate molecules based on two major selection criteria; (1) that they inhibit fibroblasts signaling pathways, (2) that they activate neural signaling pathways. According to criteria, we found a cocktail of seven small molecules (LDN193189, SB431542, CHIR99021, VPA, DAPT, Shh, and Purmorphamine) capable of directly reprogramming MEFs into NSCs. For initial examination, we performed seven small molecules together to MEFs cultures, but massive cell death was observed after drug treatment. To reduce cell death, we applied different small molecules at different time points to reprogram cells. SB431542 is an inhibitor of TGF-β receptors, and has been reported that it could enhance reprogramming in the MET transition (Ichida et al., 2009; Li et al., 2010). LDN193189 is an inhibitor of bone morphogenetic protein (BMP) receptors and helpful to enhance neural induction. For more reprogramming efficiency, it was often combined with SB431542 to facilitate neural transition (Lee et al., 2015; Mirakhori et al., 2015; Park et al., 2017). CHIR99021, an inhibitor of GSK3β, was commonly used in reprogramming studies due to its high reprogramming efficiency in combination with other small molecules (Cheng et al., 2014; Han et al., 2016; Zhang et al., 2016). DAPT is a γ-secretase inhibitor that interferes with Notch activity and promotes neural differentiation (Borghese et al., 2010). As mentioned, VPA enhance reprogramming efficiency and neuronal differentiation from NSCs. Both Shh and Purmorphamine are agonists for the Shh signaling pathway to complete the reprogramming MEFs into NSC-like cells (Moon et al., 2011). This staged-induction program was illustrated in Figure 1A.

Mouse embryonic fibroblasts in our cultures were immunostained for fibroblast markers Col1a1 with no NSCs markers like Nestin and Sox2 (Supplementary Figure S1A). It was reported that neural precursors could be isolated from juvenile and adult rodent skin (Toma et al., 2001). To eliminate the possibility of neural precursors from mouse skin, MEFs were negative to Sox2 and Nestin before being used for induction. Using seven small molecules for staged-induction, MEFs in induction medium started to aggregate on day 2, then gradually developed several colonies (Supplementary Figure S1B). MEFs expressed the fibroblast markers Collagen IV without NSCs markers Nestin (Figure 1B). SMSINS cells attaching on PDL/L was detected with typical NSCs markers Nestin and Sox2 (Figure 1C). In order to compare the characteristics, native NSC cells were isolated from embryonic mouse brain. To further detect the expression of Nestin and Sox2, we showed the analysis of FACS results for SMSINS cells and NSCs, demonstrating that Nestin-positive cells of NSCs were slightly higher than SMSINS, but Sox2-positive cells were slightly lower than SMSINS (Figure 1D).

### SMSINS Cells Performs the Characteristics of NSCs

**FIGURE 2:**
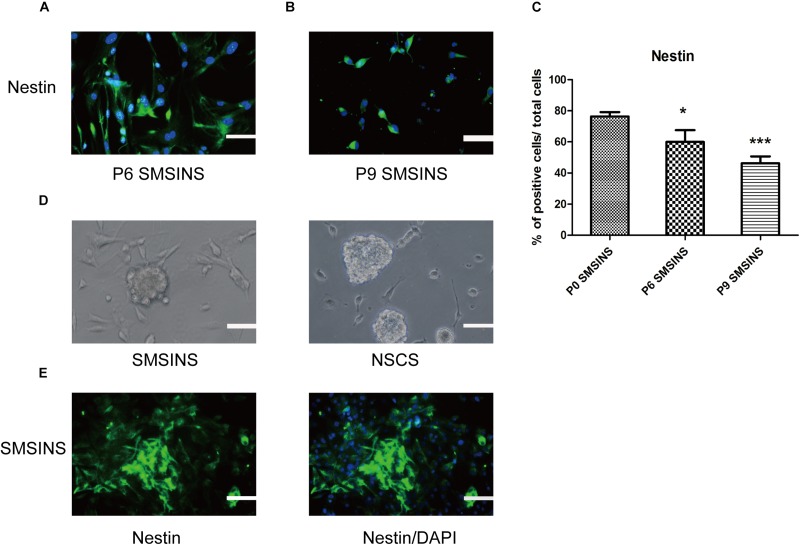
SMSINS expressed NSC markers similar to NSCs. **(A)** Neuroepithelial-like colonies of SMSINS at passage 6 (P6) stained with a specific antibody against the NSC marker Nestin (Green). **(B)** Immunostaining of Nestin (Green) in SMSINS at passage 9 (P9). Nuclei were counterstained with DAPI (blue). **(C)** Quantitative analyses of the protein expression level of Nestin in SMSINS at passage 0, 6, and 9. Data represent the mean ± SEM. *F* = 12.39 ^∗^*p* < 0.05; ^∗∗∗^*p* < 0.001 compared with P0 SMSINS; One-way ANOVA followed with Dunnett’s T3 test. **(D)** SMSINS and native NSCs formed neural spheres when cultured in suspension. **(E)** Neural spheres of SMSINS stained with specific antibodies against Nestin (Green). Nuclei were counterstained with DAPI (blue). Scale bars: 100 μm.

We dissociated SMSINS cells into single cells, and then cultured them on PDL/L plate in NSC medium, which was recorded as passage one. SMSINS cells were able to expand six passages (Figure 2A). Thereafter, SMSINS cells continued amplification and detected Nestin expression at the sixth and ninth passage (Figures 2B,C). To further detect morphological characteristics of SMSINS cells, cell colonies were dissociated into single cells. We seeded them on uncoated plates, and single cells grew neural spheres like a typical morphology of native NSCs (Figure 2D). In addition, we fixed these spheres and immunostained with Nestin to confirm them (Figure 2E).

### SMSINS Have a Potential of Multi-Potent Differentiation Into Neural Cell Lineages

**FIGURE 3:**
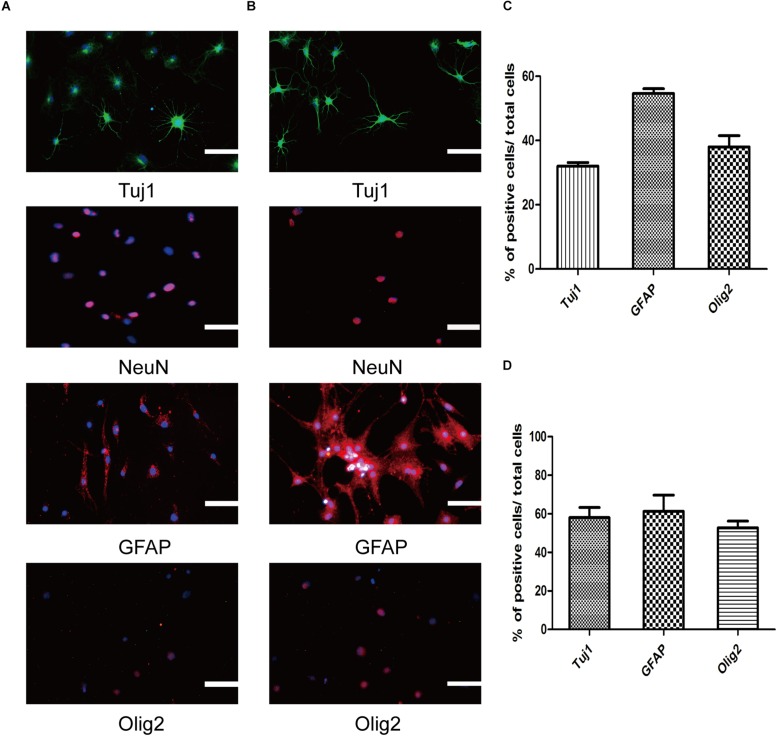
Three neural lineages differentiation potential of SMSINS *in vitro*. **(A)** Differentiation potential of SMSINS without growth factors, identified by immunostaining with antibodies against Tuj1 (Green), NeuN (Red), GFAP (Red), and Olig2 (Red). Nuclei were counterstained with DAPI (blue). **(B)** Specific differentiation of neurons, astrocytes, and oligodendrocytes from SMSINS, as determined by immunostaining with antibodies against Tuj1 (Green), NeuN (Red), GFAP (Red), and Olig2 (Red). Nuclei were counterstained with DAPI (blue). **(C,D)** Quantification of neurons, astrocytes, and oligodendrocytes differentiated from SMSINS. All data are represented as the mean ± SEM. Scale bars: 100 μm **(A,B)**.

One of the most prominent parts of our research is identifying the SMSINS cells capacity to differentiate into neural cell lineages and neuronal subtypes *in vitro*. First, SMSINS cells could spontaneously differentiate into neurons (Tuj1-positive cells, 32 ± 2%), astrocytes (GFAP-positive cells, 55 ± 3%) and oligodendrocytes (Olig2-positive cells, 38 ± 6%) by treatment with NSC medium without EGF and bFGF (Figures 3A,C). For guided differentiation into three neural lineages, SMSINS cells were cultured in the respective specific medium. As for neuron differentiation, 58 ± 9% of cells was Tuj1-positive neurons according to the neuronal protocol (Figures 3B,D). Moreover, our study proved that SMSINS cells were able to express a mature neuronal marker NeuN cultured with mature neuron medium (Figures 3A,B). Astrocytes were differentiated from SMSINS cells and 61 ± 14% of cells expressed GFAP-positive in specific differentiation morphology. In characteristic protocol, an average 53 ± 6% of cells were Olig2-positive oligodendrocytes (Figures 3B,D).

**FIGURE 4:**
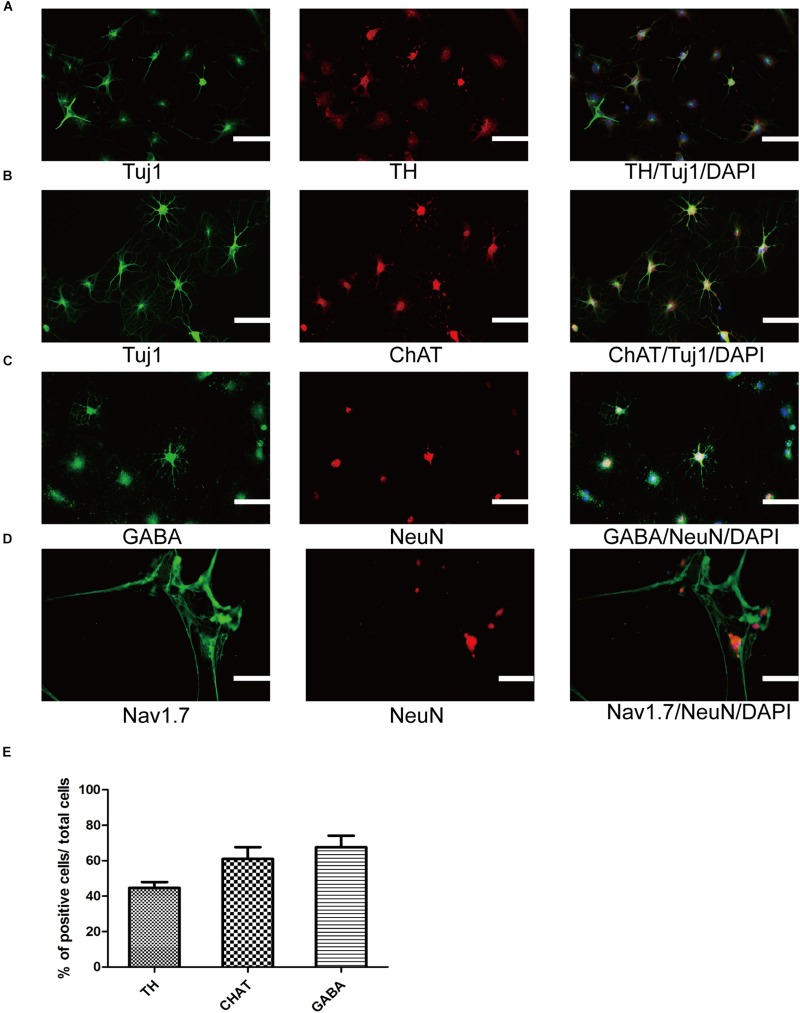
Differentiation and maturation of functional neurons from SMSINS. **(A–C)** Immunostaining of TH (Red), ChAT (Red), GABA (Green), Tuj1 (Green), and NeuN (Red), using antibodies labeled underneath the image. Nuclei were counterstained with DAPI (blue). **(D)** Sodium channels determined by immunostaining with Nav1.7 (Green). Nuclei were counterstained with DAPI (blue). **(E)** Quantification of TH dopaminergic neurons, ChAT cholinergic neurons and GABA GABAergic neurons differentiated from SMSINS. Data are represented as the mean ± SEM. Scale bars: 100 μm **(A–D)**.

To further ensure that SMSINS cells were able to differentiate into several subtype neurons *in vitro*, we detected dopaminergic, GABAergic and cholinergic neuron markers TH, GABA, and ChAT in their respective differentiation conditions (Figures 4A–C). The differentiation subtype neurons were immunostained both Tuj1 or NeuN and respective markers, and double positive cells were recorded; 45 ± 6, 61 ± 12, and 68 ± 11% of cells were positive for Tuj1/TH, Tuj1/ChAT, and NeuN/GABA (Figure 4E). To further confirm the mature functional neuron, we examined sodium channels Nav1.7 expressed in neuron (Figure 4D).

### Expression of Endogenous Relative Transcription Factors and Epigenetic Analyses During Reprogramming Process

**FIGURE 5:**
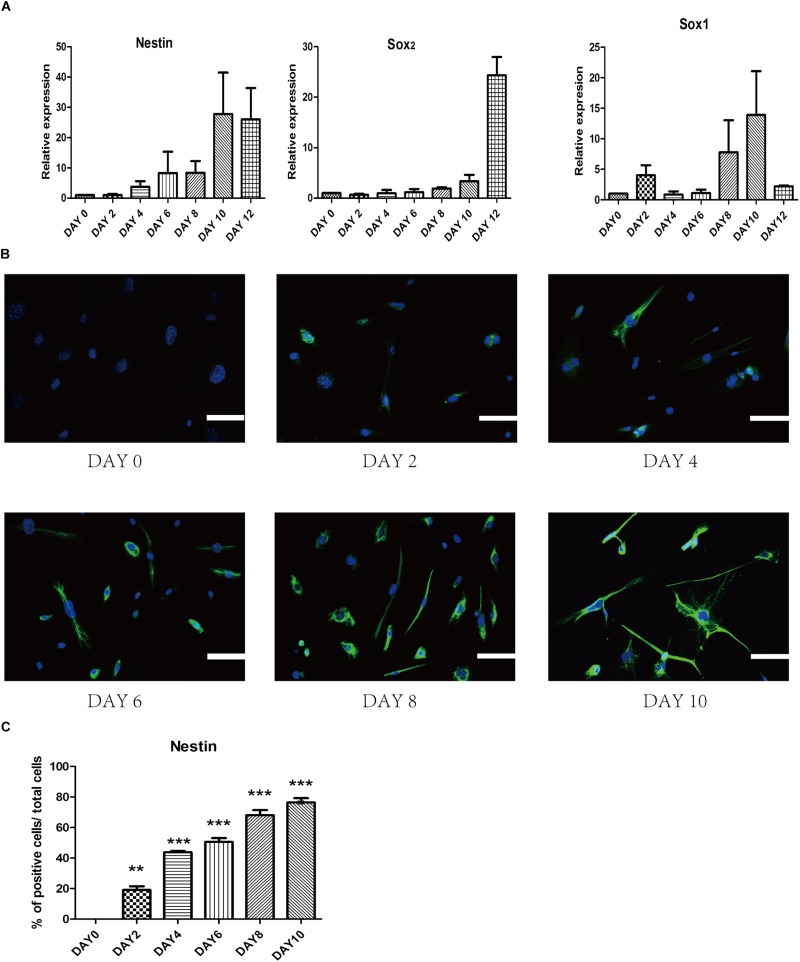
Change of endogenous relative transcription factors and expression of epigenetic analyses during the induced process. **(A)** qRT-PCR analyses of NSC markers (Nestin, Sox2, and Sox1) during the reprogramming stages. Glyceraldehyde 3-phosphate dehydrogenase (GAPDH) was used as a loading control. **(B)** Immunostaining of Nestin (Green) was detected every 2 days in the induced process. Nuclei were counterstained with DAPI (blue). **(C)** Quantitative analyses of the protein expression level of Nestin. Data represent the mean ± SEM. *F* = 97.68 ^∗∗^*p* < 0.01, ^∗∗∗^*p* < 0.001 compared with day 0; One-way ANOVA followed with Dunnett’s T3 test. Scale bars: 100 μm.

To investigate gene profile changes during reprogramming process, we analyzed the expression of several typical NSCs markers such as Nestin, Sox2, and Sox1 (Figure 5A). After small-molecule induction, we found a gradual increase in the NSC-related gene. Furthermore, we detected the expression of iPSCs-related gene Oct4 to understand whether there was a pluripotent stem cell intermediate during small-molecules treatment. The level of Oct4 at each stage was never higher than NSCs group (Supplementary Figure S2A). In a conclusion, these results suggest that our staged-induction program could activate NSC-related transcription factors.

To ensure the epigenetic analyses during reprogramming process, we performed immunofluorescence staining to examine changes in the expression of several related proteins. First, we found that epigenetic expression of Nestin level was activated at day 4 and substantially increased at day 10, which suggested a dynamic reprogramming process into the NSCs state (Figures 5B,C). To ensure that SMSINS cells were directly reprogrammed from MEFs instead of re-differentiation from intermediate pluripotent cells, we found that the iPSCs marker Oct4 had not been detected during the whole reprogramming process (Supplementary Figure S2B).

### VPA Shows Highest Efficiency in Cellular Reprogramming and Neuronal Differentiation

**FIGURE 6:**
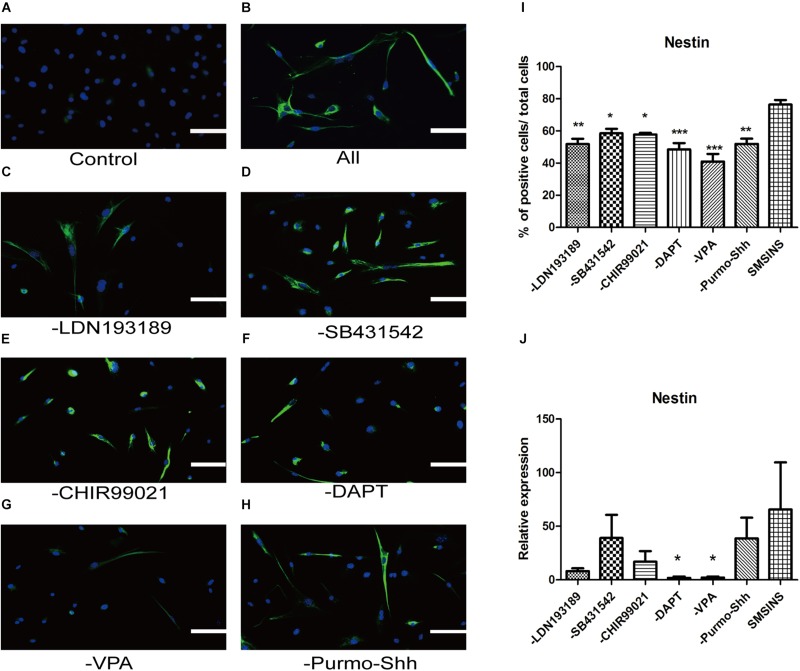
Evaluating the essential role of each small molecule during reprogramming. **(A)** MEFs treated with 1% DMSO as a control. **(B)** A staged combination of seven small molecules induced a massive number of Nestin (Green). **(C–F)** Individual removal of LDN193189 **(C)**, SB431542 **(D)**, CHIR99021 **(E)**, DAPT **(F)**, VPA **(G)**, or Purmo + Shh **(H)** from the pool of seven small molecules reduced the number of converted iNSCs. Nuclei were counterstained with DAPI (blue). Scale bars: 100 μm. **(I)** Quantitative analyses show that VPA is the most potent reprogramming factor, followed by DAPT, LDN193189, Purmo + Shh, CHIR99021 and SB431542. *F* = 8.863 ^∗^*p* < 0.05; ^∗∗^*p* < 0.01; ^∗∗∗^*p* < 0.001 compared with SMSINS; One-way ANOVA followed with Dunnett’s T3 test. **(J)** qRT-PCR analyses of removal each Individual small molecule from induced program; ^∗^*p* < 0.05; ^∗∗^*p* < 0.01; ^∗∗∗^*p* < 0.001 compared with SMSINS; Non-parametric tests was used. Data represent the mean ± SEM. GAPDH was used as a loading control.

To ascertain the precise effect of each molecule in reprogramming and optimize the staged-induction program, we withdrew one small molecule from our induction protocol. When we compared Nestin expression of drug-withdrawing groups by immunofluorescence staining and qRT-PCR analysis, we found that each group exhibited reduction of Nestin expression (Figures 6A–J). Notably, we found that both protein expression and gene level showed a significant reduction in reprogramming efficiency due to the removal of either VPA or DAPT. The reduction was even more significant with removal of VPA. Removing LDN193189 also has a significant effect in efficiency reprogramming. Interestingly, removing CHIR99021, SB431542, or Purmo + Shh slightly reduced the level of Nestin expression in qRT-PCR analysis and immunofluorescence staining assays. In order to ascertain that VPA further affected neural spontaneous differentiation, especially into neuron, we compared neuronal marker Tuj1 expression of VPA-withdrawing groups and SMSINS. Our data showed that Tuj1 expression was significantly reduced due to removal of VPA (Supplementary Figure S3).

### The Facilitative Effect of Neuronal Differentiation by VPA May Be Mediated by mTOR Signaling

**FIGURE 7:**
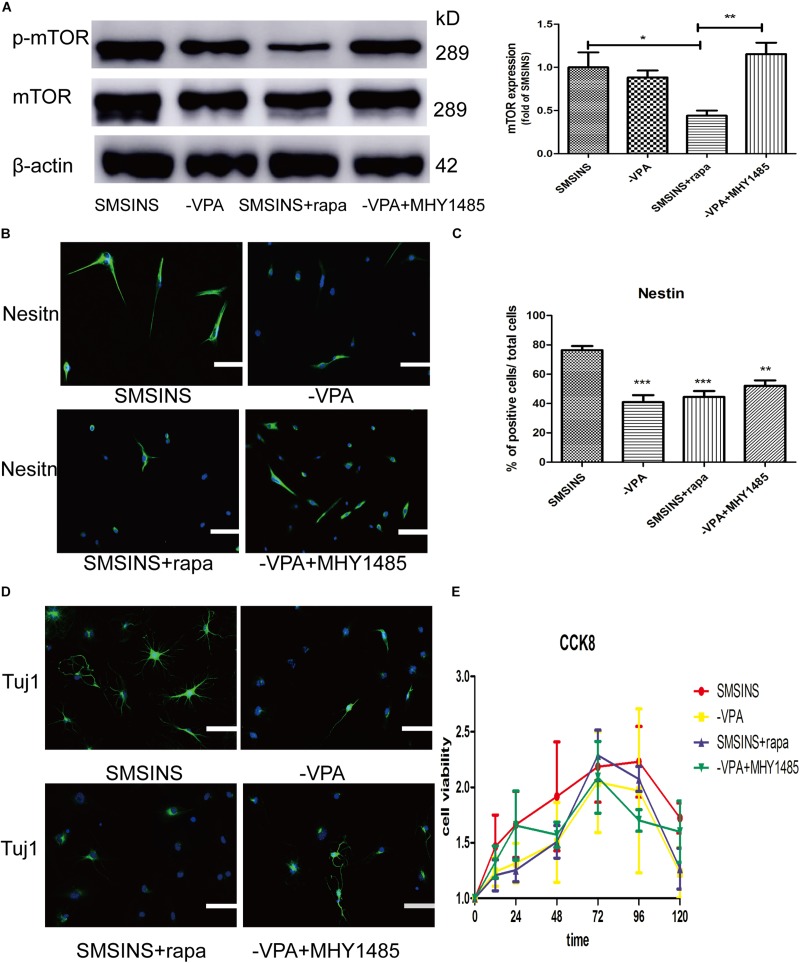
The important role of mTOR activity treated with VPA on SMSINS. **(A)** Western blots of total proteins extracted from four groups SMSINS, -VPA, SMSINS + rapamycin, and -VPA + MHY1485 using mTOR, phosphorylated mTOR and β-actin antibody. *F* = 6.566 ^∗^*p* < 0.05; ^∗∗^*p* < 0.01; ^∗∗∗^*p* < 0.001; one-way ANOVA followed with Bonferroni test. **(B)** Immunostaining of Nestin (Green) in four groups using a specify antibody. Nuclei were counterstained with DAPI (blue). **(C)** Quantitative analyses showed Nestin expression level of four groups. *F* = 13.06 ^∗^*p* < 0.05; ^∗∗^*p* < 0.01; ^∗∗∗^*p* < 0.001 compared with SMSINS; one-way ANOVA followed with Bonferroni test. *n* = 3 batches. **(D)** Neuronal differentiation potential without growth factors about four groups, as identified by immunostaining against Tuj1 (Green). Nuclei were counterstained with DAPI (blue). **(E)** Proliferative capacity of four groups was assessed by CCK-8 assay. Data represent the mean ± SEM. Scale bars: 100 μm **(B,D)**.

To detect the mechanism underlying the role of VPA in the induced stage, we evaluated the effect of mTOR inhibition rapamycin and activator MHY1485 on the induced NSCs. First, we formed four groups: SMSINS, -VPA, SMSINS + rapamycin, and -VPA + MHY1485. Then we observed the expression of mTOR of each group (Figure 7A). Secondly, we examined the effects of mTOR signaling on the induction program from the aspects of reprogramming efficiency, neuronal differentiation and proliferative potential according to the characteristics of NSC. As mentioned earlier, removal of VPA caused a significant decrease in the reprogramming efficiency. Similarly, when mTOR-specific inhibitor was added to the induction protocol, the Nestin expression was significantly reduced. Interestingly, replacing VPA with MHY1485 partially increased induction efficiency (Figures 7B,C). For detecting the differentiation effect of mTOR signaling, we caused the induced NSCs of each group to spontaneously differentiate *in vitro* (Figure 7D). We found that SMSINS cells could differentiate into neural cell lineages, but the differentiation ability of the -VPA group and the SMSINS + rapamycin group is attenuated. However, although the induction protocol was removed from VPA, supplementation with MHY1485 to activate mTOR signaling also showed neuronal phenotype.

To further confirm the proliferative potential in the different groups, we found that there was no significant difference in cell viability (Figure 7E). Interestingly, regardless of whether or not MHY1485 was added, removing VPA could slightly reduce self-renewal. Furthermore, inhibition of mTOR signaling did not result in a significant decrease in proliferative capacity. This result indicated that the mTOR signaling was independent of proliferation ability, and that the effect of VPA on proliferation was through other pathways.

## Discussion

Reprogramming somatic cells into iNSCs with the capabilities of self-renew and differentiation has great value as cell transplantation therapies in many neural diseases. Our study demonstrated that fibroblasts can be reprogrammed into NSCs by seven small molecules without involving any genetic materials. Furthermore, SMSINS cells could be passaged and differentiated into three neural lineages.

Noticeably, we reviewed previous researches and selected seven small molecules. Then, we found that adding seven small molecules together would cause cell death, which was considered to be due to the possibility of simultaneous inhibition of some signaling pathways or excessive DMSO intake. To overcome this problem, we reviewed related researches and concluded that MET should be promoted in the early induction stage (Zhang et al., 2015; Zheng et al., 2016; Liu et al., 2018). TGF-β signaling plays an important role on modulation of EMT, a process by which an epithelial cell becomes a mesenchymal cell (Grunert et al., 2003), in normal human and mouse (Valcourt et al., 2005). The generation of iNSCs from MEFs requires an increase in the MET process, through inhibition of TGF-β (Valcourt et al., 2005; Liu et al., 2018). The combination of the TGF-β inhibitor SB431542 and LDN193189 enhances the reprogramming efficiency of MET transition. Therefore, we selected the combination of the TGF-β inhibitor SB431542 and LDN193189 on early stage to enhance the reprogramming efficiency of MET transition. Then, we used CHIR99021, DAPT, and VPA to improve reprogramming efficiency, and observed that level of NSCs marker Nestin expression increased significantly. Purmorphamine/Shh promoted neural differentiation. In general, our data showed that the level of NSCs marker Nestin expression gradually enhanced during the induction period. We calculated the number of Nestin-positive cells in the passage 6 and passage 9 that was gradual decrease, which was considered the continued differentiation at each passage.

To further identify the effect of each small molecule in reprogramming by withdrawing individual molecules, we ultimately found that VPA played the most significant role in the induction protocol, followed by DAPT, LDN193189, Purmo + Shh, CHIR99021 and SB431542. VPA, a HDACs inhibitor, was widely used in reprogramming studies to eliminate the original epigenetic memory of cells. It could play important role in upregulation of ESC-specific genes and downregulation of MEFs-specific genes and improve reprogramming efficiency by more than 100-fold in iPSCs induction process, using Yamanaka factors (Huangfu et al., 2008). Our study also suggested that VPA was a key step in the induction stage. Furthermore, VPA has been shown to enhance the reprogramming efficiency of iPSC from somatic cells through the directly activated PI3K/Akt/mTOR signaling pathway to Oct4 promoter activity (Teng et al., 2014). Moreover, VPA activates the PI3K/Akt/mTOR pathway to ameliorate pathology of Duchenne Muscular Dystrophy in mouse and induce neuronal differentiation of NSCs (Gurpur et al., 2009; Zhang et al., 2017). To test whether VPA enhanced neural induction efficiency through mTOR signaling, NSCs phenotype, neuronal differentiation and self-renewal were examined using an induction protocol supplemented with mTOR-specific inhibition or activator. Our study showed that inhibiting mTOR signaling pathways without removing VPA supplement significantly decreased the induction of NSCs and neuronal differentiation. Although other studies focused on other reprogramming-related pathways of VPA such as inhibiting HDACs or activating canonical Wnt signaling (Ciani and Salinas, 2005), our study proved the importance of mTOR signaling for reprogramming efficiency and neuronal differentiation. However, the use of mTOR-specific activators instead of VPA did not fully compensate for the lack of induction efficiency. We considered that the MHY1485 was added on day 3–4, the same point time as the VPA treatment to make the experimental condition and results more comparable. The short duration of MHY1485 exposure might explain why it was not able to significantly change mTOR expression to activate the iNSCs. Moreover, VPA possibly effects the induction phase through pathways other than the activation of mTOR signaling. Overall our results suggest that mTOR signaling plays an important role in the inducing program and merits further study. Several reports showed that the mTOR pathway was a key regulator of cell growth and proliferation (Endo et al., 2009; Kaeberlein, 2013; Qin et al., 2016). However, our results demonstrated that the mTOR signaling had no significant effect on cell proliferation in three replicate experiments. In stem cells, mTOR finely regulated the balance between self-renewal and differentiation (Zhang et al., 2017). Therefore, the role of active mTOR signaling during induction is more likely to induce NSCs and neuronal differentiation.

**FIGURE 8:**
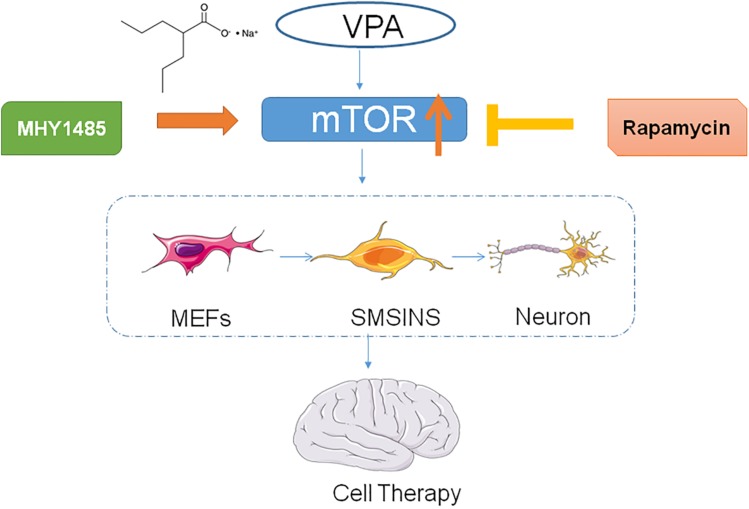
Table of contents graphic.

In conclusion, we illustrated that a small molecule stage-induction program could obtain iNSCs with the characteristics of self-renewal and neuronal differentiation. The advantage of this program was that it not only reduces the number of cell death, but also enhances efficiency without going through the pluripotent stage. Furthermore, VPA significantly improved reprogramming efficiency and neuronal differentiation. Our results suggest that VPA may stimulate mTOR signaling as conceptually illustrated in Figure 8, an aspect which we will investigate in future studies.

## Data Availability

All datasets generated for this study are included in the manuscript and/or the Supplementary Files.

## Author Contributions

QD, SL, and YG conceived and designed the study. QD, XW, and JC performed the research. QD collected and assembled the data. QD, SL, ST, and YG analyzed and interpreted the data. QD and GS wrote the manuscript. All authors approved the final version of the manuscript.

## Conflict of Interest Statement

The authors declare that the research was conducted in the absence of any commercial or financial relationships that could be construed as a potential conflict of interest.

## Abbreviations

cAMP: adenosine 3 ′,5 ′ -cyclic monophosphate
ChAT: acetyltransferase
CNS: central nervous system
DMSO: dimethyl sulfoxide
EMT: epithelial-mesenchymal transition
ESCs: embryonic stem cells
GABA: γ-aminobutyric acid
GSK3 β: glycogen synthase kinase 3 β
HDACs: histone deacetylases
iNSCs: induced neural stem cells
iPSCs: induced pluripotent stem cells
MEFs: mouse embryonic fibroblasts
MET: mesenchymal-to-epithelial transition
mTOR: mammalian target of rapamycin
NPCs: neural progenitor cells
NSCs: neural stem cells
PDL/L: poly-D-lysine/laminin
qRT-PCR: quantitative real-time PCR
Shh: sonic hedgehog
SMSINS: small-molecules staged-induction neural stem
TGF- β: transforming growth factor- β
TH: tyrosine hydroxylase
Tuj1: β-III -tubulin
VPA: valproic acid.
