# Supplementary figures and images for: Valproic Acid Enhances Reprogramming Efficiency and Neuronal Differentiation on Small Molecules Staged-Induction Neural Stem Cells: Suggested Role of mTOR Signaling
Source: Front Neurosci. 2019 Sep 4;13:867. doi: 10.3389/fnins.2019.00867 (PMC6737087; doi:10.3389/fnins.2019.00867)

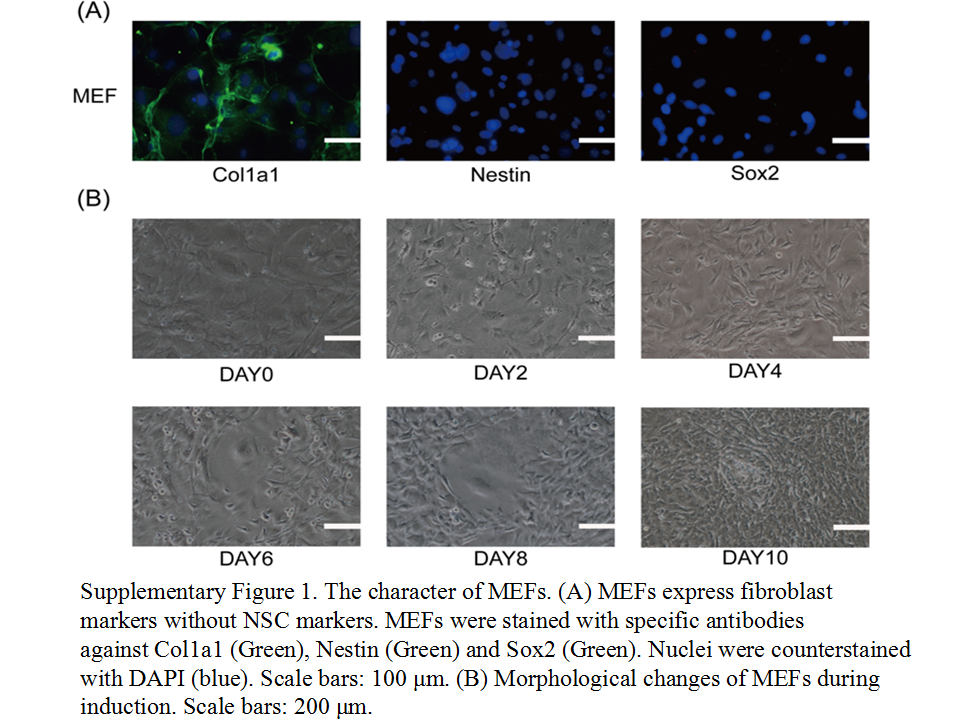

Supplement: Supplementary file 1 [file Image_1.tif]

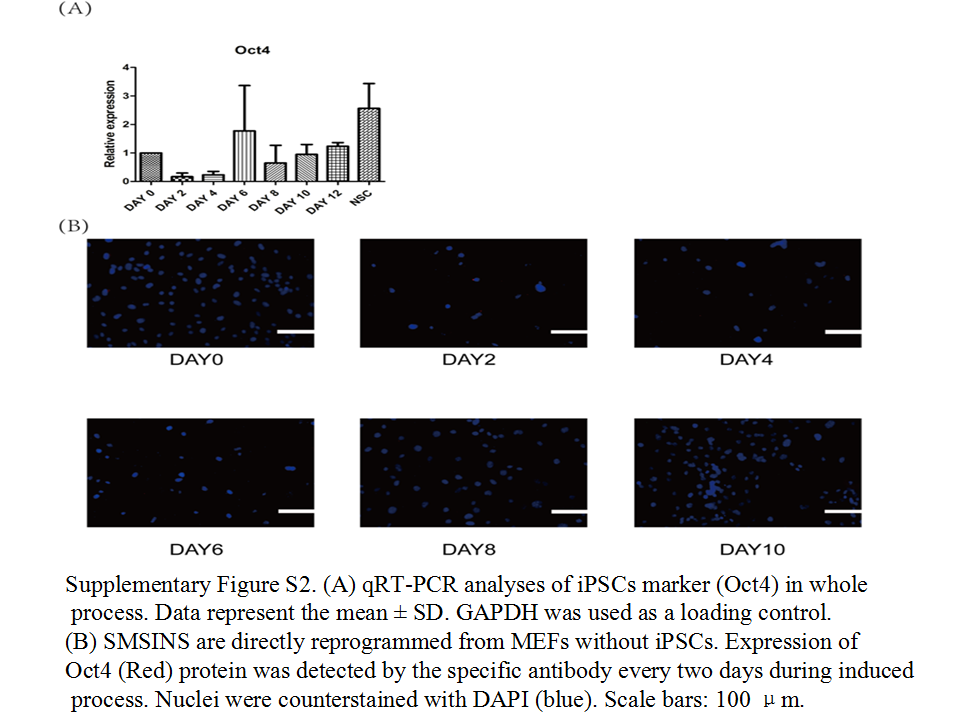

Supplement: Supplementary file 2 [file Image_2.tif]

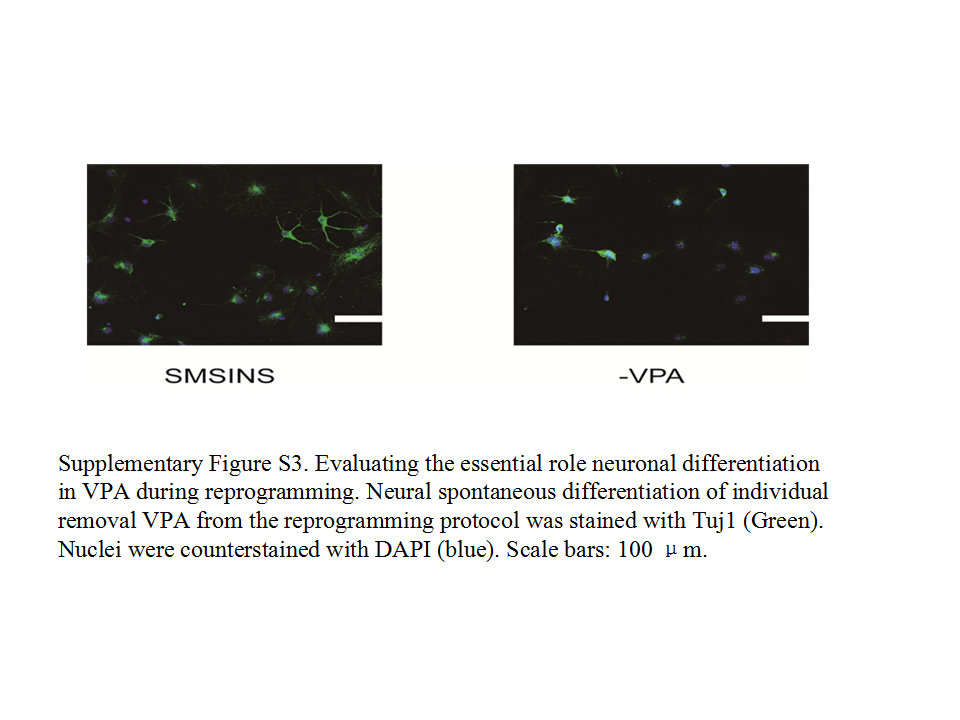

Supplement: Supplementary file 3 [file Image_3.tif]
