# Supplementary material for: Valproic Acid Enhances Reprogramming Efficiency and Neuronal Differentiation on Small Molecules Staged-Induction Neural Stem Cells: Suggested Role of mTOR Signaling
Source: Front Neurosci. 2019 Sep 4;13:867. doi: 10.3389/fnins.2019.00867 (PMC6737087; doi:10.3389/fnins.2019.00867)
Supplement: Supplementary file 4 [file Table_1.pdf]

Supplementary Table 1. Primer Information

Gene	Primer sequence (5'-3')	
GAPDH	Forward	acttcaacagcaactcccactc
	Reverse	taggcccctcctgttattatgg
Nestin	Forward	ggcatccctgaattacccaa
	Reverse	agctcatgggcatctgtcaa
Sox2	Forward	tctgtggtcaagtccgaggc
	Reverse	ttctccagttcgcagtccag
Oct4	Forward	ccaacgagaagagtatgaggc
	Reverse	caaaatgatgagtgacagacagg
Sox1	Forward	aacggagacttcgagccgacaa
	Reverse	accacttgccaaagaggccgat
